# *Ectropis obliqua*-Induced Secondary Metabolites Are Regulated by Methyl Jasmonate in a Threshold-Dependent Manner

**DOI:** 10.3390/ijms26094248

**Published:** 2025-04-29

**Authors:** Yongchen Yu, Xiaona Qian, Xiwang Li, Zhichao Chai, Dejiang Ni, Xiaoling Sun

**Affiliations:** 1 Tea Research Institute, Chinese Academy of Agricultural Sciences, Hangzhou 310008, China; yuyongchen@tricaas.com (Y.Y.); qianxiaona@tricaas.com (X.Q.); lixiwang0392@tricaas.com (X.L.); 17814680074@163.com (Z.C.); 2 Key Laboratory of Biology, Genetics and Breeding of Special Economic Animals and Plants, Ministry of Agriculture and Rural Affairs, Hangzhou 310008, China; 3 Key Laboratory of Horticulture Plant Biology, College of Horticulture & Forestry Sciences, Huazhong Agricultural University, Ministry of Education, Wuhan 430070, China

**Keywords:** methyl jasmonate, tea plant resistance, special metabolites, D-allose, *Ectropis obliqua*

## Abstract

The jasmonic acid (JA) signaling pathway has been demonstrated to play a crucial role in plant defense against herbivorous insects. However, the relationship between *Ectropis obliqua*-induced defensive metabolites and the JA signaling pathway in tea plants remains poorly understood. In this study, we investigated seven key special metabolites, including *p*-coumaroylputrescine, feruloylputrescine, prunin, naringenin, and three monolignols, to address this knowledge gap. Epicatechin was selected as a positive control based on its well-documented regulation through the JA signaling pathway. Notably, the content of all selected compounds was significantly increased by *E. obliqua* infestation. Furthermore, exogenous application of high-dose methyl jasmonate (MeJA) induced the accumulation of six of the eight compounds, excluding *p*-coumaryl alcohol and sinapyl alcohol, whereas low-dose MeJA failed to elicit their accumulation. To confirm the results, we screened two bioactive molecules, D-allose and L-theanine, which significantly increased the endogenous JA levels at low concentrations. Interestingly, neither D-allose nor L-theanine triggered the biosynthesis of these defensive compounds. Additionally, D-allose-treated tea leaves had no significant effect on the performance of *E. obliqua* larvae. These findings demonstrate that the metabolic accumulation induced by *E. obliqua* is mediated through a high-threshold JA signaling cascade. This study provides novel insights into the relationship between plant resistance and JA signaling pathway, advancing our understanding of special metabolites mediated plant-insect interactions.

## 1. Introduction

In nature, plants have evolved multilayered defense strategies to counteract herbivorous insects, encompassing both constitutive and inducible plant resistance [1,2]. The induced plant resistance is initiated by damage-associated molecular patterns (DAMPs) and herbivore-associated molecular patterns (HAMPs) [3,4]. The initial recognition triggers early signal transductions characterized as cytosolic calcium influx, reactive oxygen species bursts, and plasma membrane depolarization [4,5]. These early signaling events subsequently trigger mitogen-activated protein kinase cascades, which orchestrate the downstream phytohormonal networks. Jasmonic acid (JA), salicylic acid (SA), abscisic acid (ABA), and ethylene (ET) constitute the four major phytohormones induced by herbivory, which engage in complex crosstalk to fine-tune plant defense responses through coordinated transcriptional reprogramming and metabolic reconfiguration [6,7].

Of note, jasmonates (JAs), mainly including jasmonic acid (JA), its methyl ester methyl jasmonate (MeJA), and its amino acid conjugate jasmonoyl-L-isoleucine (JA-Ile), dominate the induced plant defense against chewing herbivores [4,5]. MeJA has been widely employed as an effective elicitor of plant resistance across various species, primarily due to its superior induced efficacy compared to JA [8,9,10]. The role of MeJA in inducing plant resistance to herbivores has been widely demonstrated in diverse plant–herbivore study systems. For instance, in pepper (*Capsicum annuum*), exogenous MeJA application significantly suppresses *Myzus persicae* fecundity while paradoxically extending its oviposition period compared to untreated controls [11]. Similarly, exogenous application of MeJA enhances tobacco (*Nicotiana tabacum*) resistance to both whitefly (*Bemisia tabaci*) and cotton bollworm (*Helicoverpa armigera*) [12]. The detrimental effects of MeJA-treated plants on herbivore performance are attributed to induced defense responses, characterized by enhanced defense-related enzyme activities, increased emission of volatile organic compounds (VOCs), and accumulation of phytotoxic secondary metabolites [4].

Herbivore infestation induces a significant reconfiguration of plant secondary metabolites, many of which are hypothesized to function as defensive compounds through their toxic or deterrent properties [13,14]. The biosynthesis of substantial defensive metabolites has been verified to be regulated by JA signaling pathway. According to chemical structures, these metabolites can be classified into four major groups: terpenoids, phenolics, nitrogen-containing compounds, and sulfur-containing compounds [15]. Volatile compounds function in deterring the growth rate of herbivores, repelling herbivore moths, attracting natural enemies of herbivores, and priming neighbor plants, while non-volatile metabolites act as deterrents or toxicants to defense against herbivorous insects [13,16]. Non-volatile metabolites, including phenolamides, benzoxazinoids, nicotine, glucosinolates, and phenolic compounds, have been precisely studied in plant defense against herbivores mediated by JA signaling pathway [17,18,19,20]. For instance, *Arabidopsis thaliana* with an impaired JA signaling pathway exhibits a lower content of glucosinolates and compromised plant defense against the generalist herbivore *Spodoptera littoralis* [20]. Similarly, MYC2-VIGS *Nicotiana attenuate* plants show inhibited nicotine content and suppressed resistance to *Mandca sexta* larvae [21]. In addition, metabolites from the phenylpropanoid pathway also contribute to plant defense against herbivores. For instance, *Nilaparvate lugens* infestation induced the accumulation of sakuranetin in rice (*Oryza sativa*) depending on the JA signaling pathway, which leads to high mortality of *N. lugens* nymphs [22]. Therefore, a comprehensive investigation of herbivore-induced plant responses is essential, encompassing three aspects: (1) the characterization of special metabolite profiles under herbivore infestation, (2) the elucidation of molecular mechanisms underlying the biosynthesis of herbivore-induced metabolites, and (3) the functional analysis of these induced compounds. Such systematic research will provide valuable insights for developing innovative pest control strategies.

Tea plants (*Camellia sinensis*), rich in secondary metabolites, confront various herbivores in tea plantations. Numerous studies have demonstrated that the JA signaling pathway plays a crucial role in defending against the tea geometrid, *Ectropis obliqua* [23,24,25,26,27,28,29]. To elaborate, as to the fact that *E. obliqua* infestation induces the burst of JAs, the application of elicitors such as gallic acid and JA-Ile macrolactone 5b enhances tea plants’ resistance to *E. obliqua* larvae, while JA pathway inhibitors, such as Lyn3, DIECA, and salicylhydroxamic acid (SHAM), significantly enhance tea plants’ susceptibility to *E. obliqua* larvae [24,25,26,29]. Recent studies have systemically investigated the role of MeJA in modulating tea flavor and aroma profiles, and MeJA has been found to induce the emission of diverse volatile organic compounds (VOCs) in a dose-dependent manner as well [10,30]. However, the relationship between MeJA-mediated and *E. obliqua*-induced metabolic reconfiguration remains poorly characterized. Since tea leaves gradually accumulate JA levels during *E. obliqua* infestation, a critical knowledge gap persists in establishing quantitative relationships between metabolite accumulation and JA levels. Thus, exogenous application of MeJA would precisely control the stress intensity through standardized dosage application, thereby facilitating systemic investigation of the metabolite reconfiguration during *E. obliqua* infestation. Alternatively, due to the limitations of the genetic transformation system of tea plants, chemicals with similar functions to jasmonates are needed to better explain tea plant resistance mechanisms.

In this study, we employed a systematic approach to investigate the relationship between the accumulation of special metabolites elicited by *E. obliqua* and exogenous application of MeJA in different doses, along with screening chemicals with similar functions to MeJA. First, we identified and characterized *E. obliqua*-induced metabolites. Subsequently, we validated that the accumulation of these special metabolites could be regulated by the exogenous application of MeJA in high doses. Specific chemicals, such as D-allulose, D-allose, γ-aminobutyric acid (GABA), and L-theanine, were applied exogenously to screen potential elicitors through measuring JA content. Finally, we found that D-allose elicited a low yet statistically significant induction of JAs, which failed to trigger the subsequent accumulation of special metabolites or enhance herbivore resistance. Our results significantly enhance the comprehensive understanding of the relationship between the JA signaling cascade and *E. obliqua*-induced metabolic reconfiguration in tea plants.

## 2. Results

### 2.1. E. obliqua Infestation Alters the Metabolic Profile of Tea Plants

**Figure 1:**
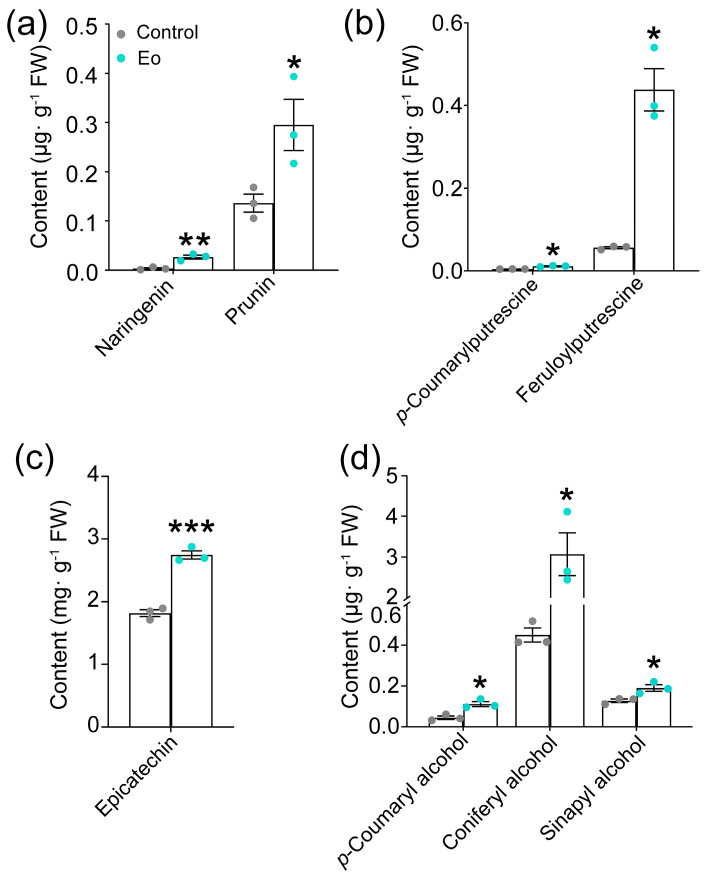
*Ectropis obliqua* infestation modulated the accumulation of eight metabolites in tea plants. (**a**) Flavonoids; (**b**) phenolamides; (**c**) epicatechin; (**d**) monolignols. The asterisk indicates a significant difference between treatment and control (* *p* < 0.05, ** *p* < 0.01, *** *p* < 0.001, Student’s *t*-test, *n* = 3).

Catechin (C), epicatechin (EC), epigallocatechin gallate (EGC), epicatechin gallate (EGCG), quercetin-3-O-glucoside, naringenin, prunin, *p*-coumaroylputrescine, feruloylputrescine, and monolignols have been reported to be induced under biotic and abiotic stresses and might be induced by JAs in tea plants [23,29,31,32,33,34]. Thus, they were selected to investigate the metabolic profile upon *E. obliqua* infestation and exogenous application of MeJA, and EC was used as the positive control (Figure 1). Intriguingly, the content of naringenin (7.78-fold), prunin, and EC, along with phenolamides including *p*-coumaroylputrescine (2.53-fold) and feruloylputrescine (7.84-fold), was significantly upregulated upon *E. obliqua* infestation (Figure 1a–c). Similarly, three monolignols were significantly induced as well, especially coniferyl alcohol, which increased by 6.82-fold (Figure 1d). These results demonstrated that all target metabolites were positively elicited by *E. obliqua* infestation.

### 2.2. Effect of Exogenous Application of MeJA on E. obliqua-Induced Key Metabolites

**Figure 2:**
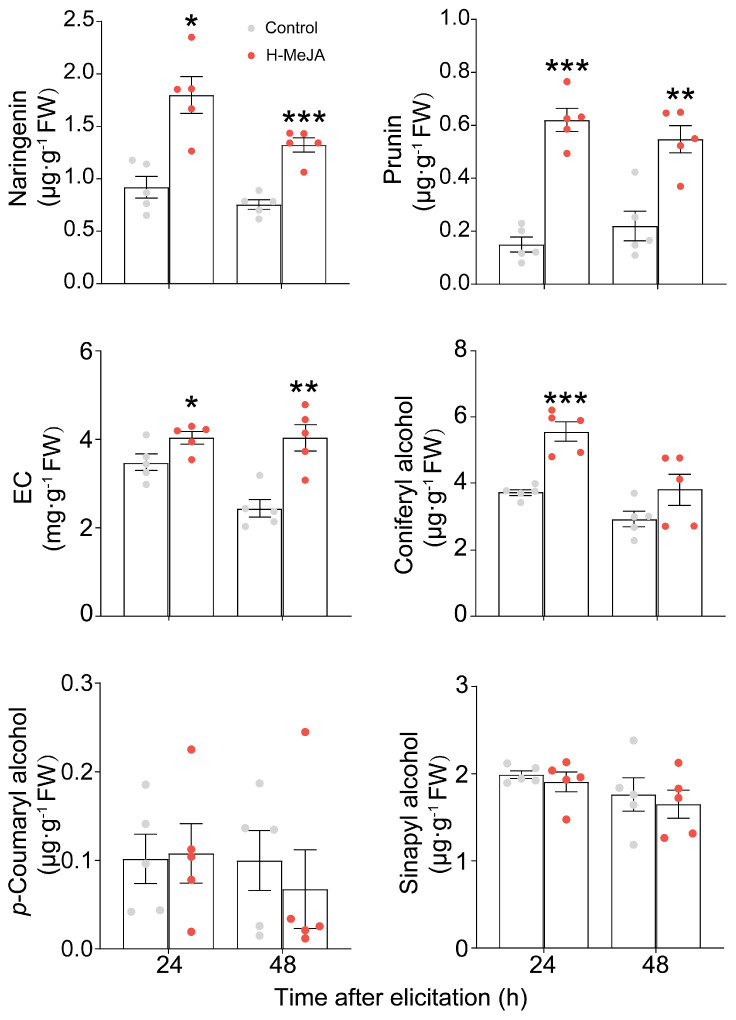
Exogenous application of a high dose of MeJA (H-MeJA) modified most of the metabolic profile elicited by *Ectropis obliqua* infestation (*n* = 5). The asterisk represents a significant difference between treatment and control (* *p* < 0.05, ** *p* < 0.01, *** *p* < 0.001, Student’s *t*-test, *n* = 5).

**Figure 3:**
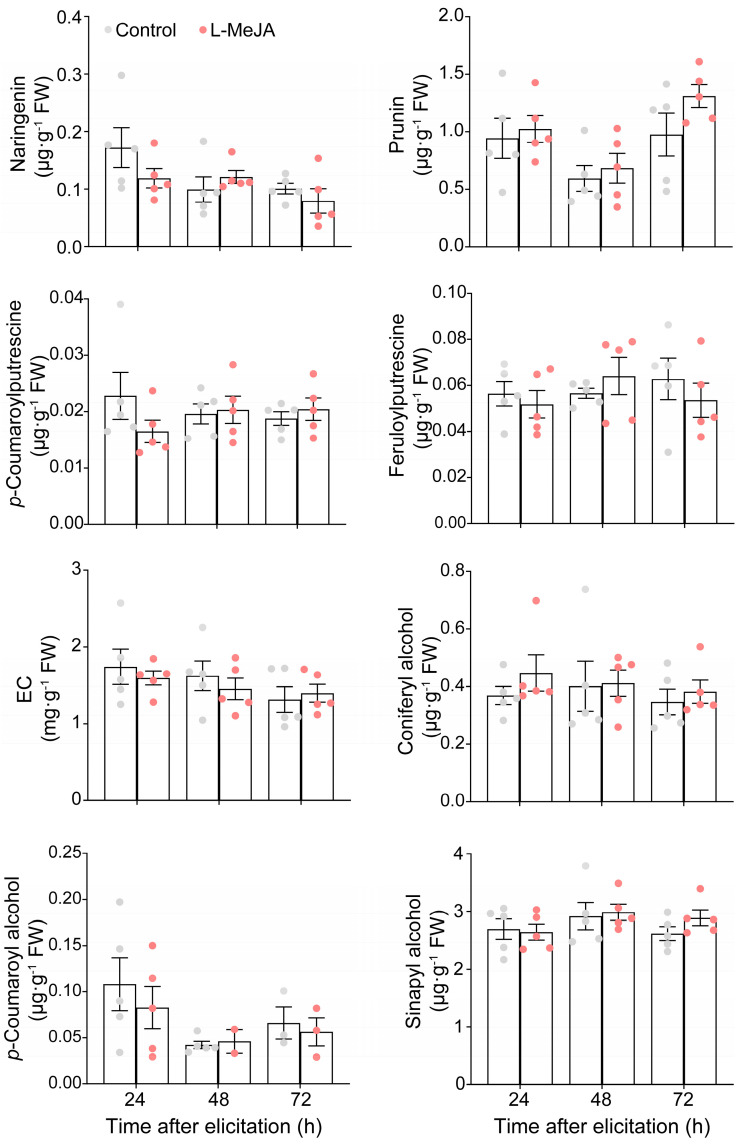
Exogenous application of a low dose of MeJA (L-MeJA) did not modify the metabolite profile elicited by *Ectropis obliqua* infestation.

*E. obliqua* infestation significantly induced the synthesis of JAs [23]; thus, exogenous application of MeJA is an effective way to decipher the JA-mediated secondary metabolites. Exogenous application of 25 mL of 1 mM MeJA per plant significantly induced the accumulation of naringenin, prunin, and EC at 24 h and 48 h post application of MeJA (Figure 2). Moreover, the content of coniferyl alcohol was also enhanced at 24 h post treatment (Figure 2). However, the content of *p*-coumaryl alcohol and sinapyl alcohol was unchanged (Figure 2). The content of *p*-coumaroylputrescine and feruloylputrescine was also significantly increased at 24 h and 48 h after 25 mL of 1 mM MeJA elicitation (submitted). However, the exogenous application of 8 mL of 1 mM MeJA per tea plant showed no elicited effect on the eight metabolites (Figure 3).

### 2.3. D-Allose and L-Theanine Induced Statistically Significant but Quantitatively Limited Increases in JA Levels

**Figure 4:**
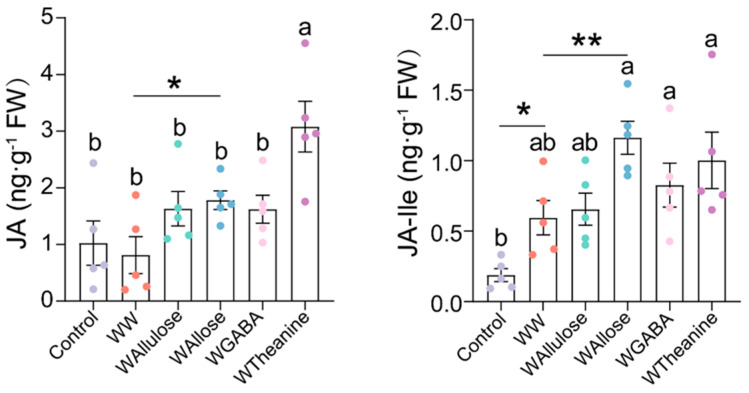
Mean content (±SE) of jasmonic acid (JA) and jasmonoyl-isoleucine (JA-Ile) elicited by potential elicitors in *Camellia sinensis*. Control: intact tea plant; WW: mechanical damage plus deionized water; WAllulose: mechanical damage plus D-allulose; WAllose: mechanical damage plus D-allose; WGABA: mechanical damage plus GABA; WTheanine: mechanical damage plus L-theanine. Different letters mean significant differences among treatments (*p* < 0.05, Turkey’s honest significant difference post-hoc test, *n* = 5). Asterisks indicate significant differences between two groups (* *p* < 0.05, ** *p* < 0.01, Student’s *t*-test, *n* = 5).

D-allose and D-allulose have been identified as effective elicitors to induce plant immunity against various pathogens [35,36,37,38,39]. L-theanine, a specialized amino acid in tea plants, has been demonstrated to be closely associated with JA content [40], while GABA has been verified to trigger JA accumulation in tomato plants (*Solanum lycopersicum*) and sweet orange (*Citrus sinensis*) [41,42]. Therefore, these molecules were selected to screen effective components that could induce endogenous JA cascades in tea plants. Among the candidates, D-allose treatment significantly enhanced endogenous JA and JA-Ile levels to 1.78 and 1.16 ng·g^−1^ FW, respectively, representing 2.2-fold and 1.95-fold increases over wounding plus distilled water (WW) at 6 h post-treatment. Meanwhile, L-theanine treatment significantly induced a 3.8-fold increase in JA (3.08 ng·g^−1^ FW) levels (Figure 4). However, GABA and D-allulose failed to elicit significant changes in JA and JA-Ile levels (Figure 4). Therefore, D-allose and L-theanine were selected for further study.

### 2.4. Neither D-Allose nor L-Theanine Altered the Target Metabolites

**Figure 5:**
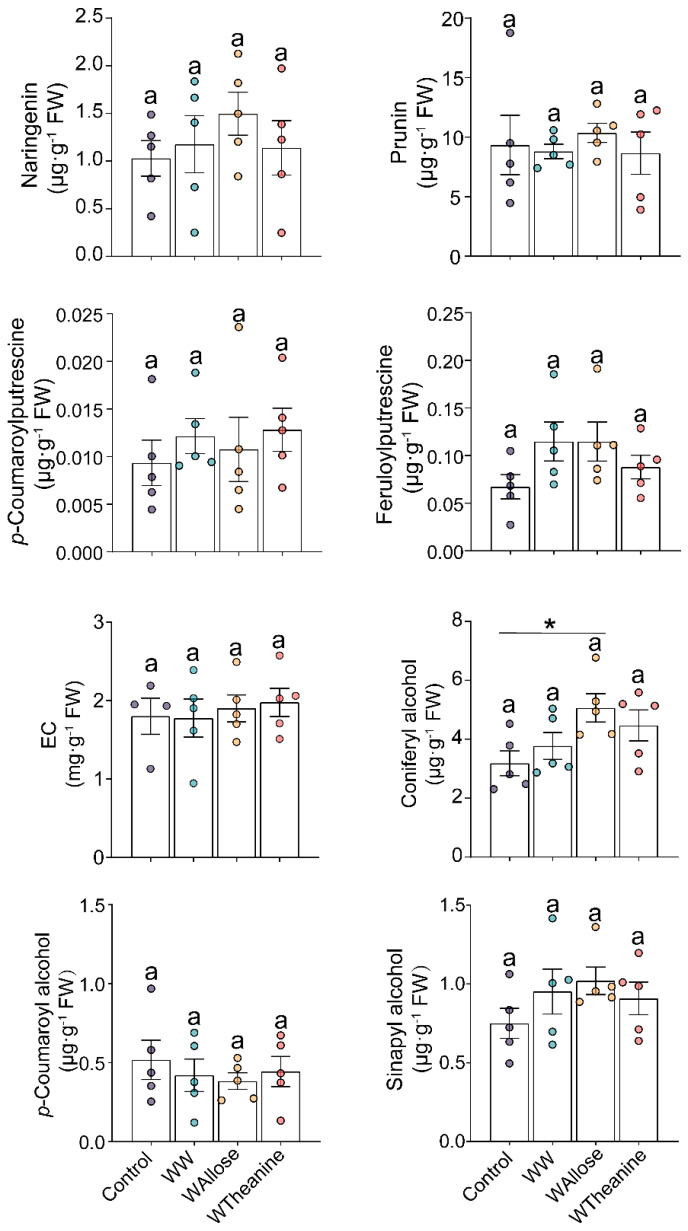
Mechanical damage plus D-allose (WAllose) and L-theanine (WTheanine) did not alter the metabolite profile elicited by *Ectropis obliqua* infestation. Letter (a) mean no significant differences among treatments (*p* > 0.05, Turkey’s honest significant difference post-hoc test, *n* = 5). The asterisk denotes a significant difference between treatment and control (* *p* < 0.05, Student’s *t*-test, *n* = 5).

Our previous study has demonstrated that simulated *E. obliqua* feeding elicits JA and JA-Ile bursts exceeding 100 and 200 ng·g^−1^ FW, respectively [23]. Both D-allose and L-theanine significantly induced JA accumulation in tea leaves, reaching concentrations of 1.78 ± 0.16 ng·g^−1^ FW and 3.08 ± 0.45 ng·g^−1^ FW, respectively (Figure 4). Interestingly, D-allose significantly elicited the accumulation of coniferyl alcohol compared to control. None of the rest of the metabolites was remarkably triggered. Moreover, L-theanine treatment did not significantly induce the accumulation of the target metabolites (Figure 5).

### 2.5. D-Allose Did Not Enhance Tea Pant Resistance to E. obliqua

**Figure 6:**
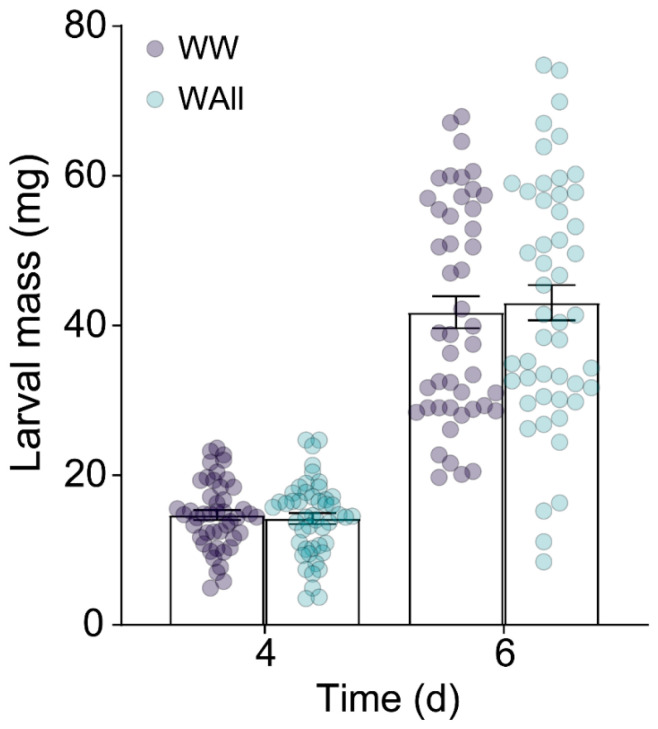
Exogenous application of D-allose did not enhance tea plant resistance to *Ectropis obliqua* larvae (*n* = 45–50). WW: mechanical damage plus deionized water; WAll: mechanical damage plus D-allose.

To investigate the potential elicitation effect of D-allose on tea plant resistance to *E. obliqua* larvae, a bioassay was conducted. Statistical analysis showed no significant differences in larval mass (*p* > 0.05) between *E. obliqua* feeding on D-allose-treated versus control plants at either 4 or 6 days post-treatment (Figure 6).

## 3. Discussion

Numerous studies have demonstrated that the JA signaling pathway triggers metabolic reconfiguration in tea plants to defend against major herbivores, including the tea green leafhopper (*Empoasca onukii*), tea aphid (*Toxoptera aurantia*), and tea geometrid (*E. obliqua*) [23,43,44,45]. For instance, infestation of tea green leafhoppers and tea geometrids triggered a rapid accumulation of JA, reaching 100 and 200 ng·g^−1^ FW [44]. Similarly, *E. obliqua* feeding induced bursts of both JA and JA-Ile, with their concentrations exceeding 200 ng·g^−1^ FW and 50 ng·g^−1^ FW, respectively [45]. Furthermore, foliar application of 2 mM MeJA (50 mL per plant) effectively induced the emission of volatile organic compounds in tea plants [10], and tea shoots soaking in 2.5 mM JA solution for 12 h significantly activated the accumulation of total catechins, while 1.0 mM of JA was ineffective [44]. Our previous study has demonstrated that mechanical wounding by a pattern wheel and simulated feeding of *E. grisescens* induced a burst of JA and JA-Ile, with concentration exceeding 100 and 200 ng·g^−1^ FW, respectively, which significantly induced the accumulation of EC and the subsequent defense against *E. grisescens* [23]. In the present study, WW, WAllose, and WTheanine induced JA accumulation to 0.81, 1.78, and 3.08 ng·g^−1^ FW, respectively, and JA-Ile accumulation to 0.59, 1.16, and 1.11 ng·g^−1^ FW, respectively (Figure 4). However, these JA and JA-Ile levels were insufficient to elicit the metabolic changes associated with *E. obliqua* infestation or confer resistance to *E. obliqua* larvae. In this study, we found that the accumulations of *p*-coumaroylputrescine, feruloylputrescine, naringenin, prunin, EC, and three monolignols were significantly induced upon *E. obliqua* infestation, of which the accumulation of *p*-coumaroylputrescine, feruloylputrescine, naringenin, prunin, coniferyl alcohol, and EC were also significantly elicited by a high dose of MeJA (Figure 1 and Figure 2). Surprisingly, the application of a low dose of MeJA resulted in no significant changes to the target metabolites (Figure 3). Moreover, two elicitors in our potential list, D-allose and L-theanine, inducing low yet remarkable JAs (Figure 4), did not induce the accumulation of the target metabolites significantly (Figure 5). Furthermore, we found D-allose-treated tea plants did not enhance tea plant resistance to *E. obliqua* larvae (Figure 6). These results revealed that both the exogenous application of a low dose of MeJA and endogenous low levels of JAs failed to induce effective metabolic changes in tea plants, indicating a fine-tuned mechanism of tea plants’ response to *E. obliqua*.

The branches of phenylpropanoid pathway-producing flavonoids, phenolamides and monolignols, regulated by the JA signaling pathway, play a pivotal role in plants’ defense against biotic stresses [46]. For example, tea aphid infestation significantly induced the accumulation of *p*-coumaryl alcohol in resistant tea cultivars, whereas no such induction was observed in susceptible cultivars; this difference is strongly associated with tea plant resistance to tea aphids [47]. Our results showed that *E. obliqua* infestation induced the accumulation of *p*-coumaryl alcohol and sinapyl alcohol (Figure 1d). However, neither a low dose nor a high dose of MeJA successfully induced their accumulation (Figure 2 and Figure 3). Similarly, previous studies have shown that exogenous application of MeJA triggered the accumulation of distinct lignin precursors; for instance, the content of coniferyl alcohol and the expression level of its biosynthetic genes were significantly upregulated in *A. thaliana* [48]. Furthermore, the accumulation of phenolic acids, such as *p*-coumaric acid and ferulic acid, exhibited a dose-dependent responsive manner to MeJA treatment, with 50 μM MeJA failing to trigger ferulic acid biosynthesis in *Brachypodium distachyonc* [49]. Notably, the majority of *E. obliqua*-induced metabolites showed consistent upregulation pattern upon high-dose MeJA treatment, while none of the target metabolites were effectively elicited by low-dose MeJA. A case study also exhibited similar results: tea plants sprayed with 50 mL of 10 mM MeJA per plant significantly elicited the emission of volatiles, characterized by both increased compositional diversity and higher quantitative levels of released volatiles compared to those emitted from tea plants treated with 50 mL of 2 mM MeJA per plant [10]. These findings collectively demonstrated that *E. obliqua*-induced metabolic reconfiguration was mediated through the JA signaling pathway in a dose-dependent manner.

A previous study demonstrated that GABA triggered slight but significant JA accumulation, resulting in a primed defense response against *Botry cinerea* in tomato plants [42]. We also found that 8 mL of 1 mM MeJA failed to incur the accumulation of eight target metabolites. Furthermore, our study revealed that both D-allose and L-theanine treatment triggered a JA cascade in tea leaves. D-allose treatment resulted in a significant JA-Ile accumulation, but L-theanine treatment failed (Figure 4). This observation aligns with previous findings in rice [35,38,39]. Interestingly, despite JA-Ile level was upregulated, D-allose treatment failed to enhance the accumulation of target metabolites and the subsequent resistance in tea plants (Figure 5 and Figure 6), suggesting that the induced low JA-Ile level was insufficient to confer enhanced resistance against herbivores. These findings suggested that although D-allose acted as an elicitor to activate JA cascade in tea plants, the level might not reach the threshold required for significant metabolic reprogramming and herbivore resistance. It is worth noting that similar results have been found in L-theanine-treated tea plants, which we will exhibit in another paper. This threshold effect could be attributed to species-specific differences in JA sensitivity or the existence of alternative regulatory mechanisms in tea plants.

In conclusion, our study demonstrates that *E. obliqua* infestation induces metabolic reconfiguration in tea plants through the JA signaling pathway, with a notably high activation threshold required for effective defense responses. We further identified D-allose as a potential elicitor capable of inducing JA accumulation. However, the induced JA levels by D-allose were insufficient to trigger significant metabolic changes or enhance herbivore resistance in tea plants, highlighting the need for precise threshold determination. Future research should focus on quantifying the minimum JA and JA-Ile concentrations required for eliciting effective defense responses, as well as exploring strategies to optimize JA signaling activation in tea plants.

## 4. Materials and Methods

### 4.1. Plants and Herbivorous Insects

Three-year-old potted tea plants (Camellia sinensis cv. ‘Longjing 43’) were cultivated under controlled environmental conditions in a growth chamber. The plants were maintained at a temperature of 26 ± 2 °C, relative humidity of 70–80%, and 12 h light/dark photoperiod. To ensure optimal growth, plants were fertilized monthly using a balanced nutrient solution and irrigated every other day. Prior to experiments, healthy tea plants with uniform morphology and growth vigor were selected. The larvae of *Ectropis obliqua* were initially collected from a tea plantation in Shaoxing, China. The herbivores were maintained in an incubator at 26 ± 2 °C, 70 ± 5% relative humidity, and a 12 h light/dark photoperiod. Fresh tea shoots from ‘Longjing 43’ cultivars in tea plantation were provided to *E. obliqua* larvae. After one generation, caterpillars reared under these standardized conditions were used for experiments.

### 4.2. E. obliqua Infestation

Following the experimental protocols established in our previous study [23], eight three-day-old *E. obliqua* larvae were starved for 4 h before being introduced onto a second set of fully expanded leaves that were immediately enclosed with a mesh sleeve. Controls were enclosed with empty mesh sleeves. After 96 h feeding, the main veins of tea leaves were ablated to diminish the uneven distribution of metabolites between leaf blades and main veins, then twelve of leaves from treatment and control of tea plants were collected and pooled into three samples for subsequent analysis.

### 4.3. MeJA Treatment

Methyl jasmonate (MeJA) was purchased from Sigma Chemical Co. (St Louis, MO, USA). One mM MeJA solution was prepared by dissolving the compound in 0.2% (*v*/*v*) methanol. For experimental treatments, two different application methods were employed: (1) low-dose treatment: 8 mL of MeJA solution was applied to each tea plant, with visible droplets on tea leaves; (2) high-dose treatment: 25 mL of MeJA solution was evenly sprayed on both adaxial and abaxial leaf surfaces of tea plants, with liquid dripping off the tea leaves. Plants treated with the same volume of 0.2% methanol were used as the controls. All treated plants were placed in a sealed transparent plastic device (70 × 70 × 70 cm) and reared in a climate chamber with temperature at 26 ± 2 °C, relative humidity at 70 ± 5%, and a photoperiod of 12 h light/dark. Fifteen leaves from each treatment were collected and pooled into five samples.

### 4.4. D-Allose, γ-Aminobutyric Acid, D-Allulose and L-Theanine Treatment

D-allose (Aladdin, Shanghai, China), D-allulose (Macklin, Shanghai, China), γ-aminobutyric acid (Aladdin), and L-theanine (Aladdin) were dissolved in distilled water, with concentrations of 0.5 mM, 0.5 mM, 1.45 mM, and 4 mM, respectively. The concentrations of γ-aminobutyric acid (GABA) and L-theanine used in present study were according to their physiological concentrations in tea aphid honeydew, while the concentrations of D-allose and D-allulose were in reference to the literature [35,36,37,38,39]. Plants treated with the same volume of distilled water were used as the controls. The second fully expanded leaf was mechanically wounded by ten insect needles, and 10 μL of distilled water was applied to the wounding site, which was repeated twice at 24 h intervals. D-allose, GABA, D-allulose (also named D-psicose), and L-theanine treatments were consistent with the method, as was the control. Samples were harvested at 6 h and 48 h post-treatment for phytohormone and flavonoid analysis, respectively. Ten leaves from each treatment were collected and pooled into five independent replicates for analysis.

### 4.5. Flavonoid Extraction and Quantification

A finely ground tea powder (0.1 g) was homogenized with 1 mL of 70% (*v*/*v*) methanol aqueous solution. The mixture was vortexed for 5 min and then subjected to thermal-assisted extraction in a thermostatic water bath at 70 °C for 20 min. After cooling to room temperature, the extract was centrifuged at 12,000× *g* for 10 min at 4 °C, and 500 μL supernatant was collected for subsequent analysis after being filtered with a 0.22 μm nylon membrane (Jin Teng, Tianjin, China). Flavonoid profiling was conducted using a Waters ACQUITY UPLC H-Class system (Milford, MA, USA) coupled with a Xevo TQ-S micro triple quadrupole mass spectrometer operated in multiple reaction monitoring (MRM) mode.

### 4.6. Phytohormone Quantification

JA and JA-Ile were quantified following the method described in our previous study [25]. The concentrations of phytohormones were determined using internal standards.

### 4.7. Herbivore Bioassay

The second leaves of control and D-allose-treated tea plants (48 h after the final treatment) were detached. Five 3-day-old *E. obliqua* larvae, starved for 8 h, were introduced onto a leaf in a square Petri dish (*n* = 45–50). The leaves were replaced when approximately 50% of the leaf area had been consumed. Larval mass was measured at 4 and 6 d post-inoculation. This experiment was documented under the same condition as those for rearing.

### 4.8. Statistical Analysis

Statistical analysis was performed using SPSS 22 software (IBM Inc., New York, NY, USA). Differences between treatment and control were assessed by Student’s *t*-test. Differences among more than three groups were determined by one-way ANOVA, and Tukey’s honest significant difference (HSD) post-hoc test was used.

## Data Availability

Data available on request from the authors.

## Abbreviations

The following abbreviations are used in this manuscript:

MeJA	Methyl jasmonate
JA	Jasmonate acid
EC	Epicatechin
GABA	γ-aminobutyric acid
